# IGA nephropathy and spinal epidural abscess after COVID-19 infection: a case report

**DOI:** 10.2217/fvl-2021-0314

**Published:** 2022-06-28

**Authors:** Burak Göre, Ezgi Coşkun Yenigün, Şimal Köksal Cevher, Emre Çankaya, Numan Aydın, Fatih Dede

**Affiliations:** ^1^Department of Internal Medicine, Ankara City Hospital, Ankara, 06800, Turkey; ^2^Department of Nephrology, Ankara City Hospital, Ankara, 06800, Turkey

**Keywords:** acute kidney injury, COVID-19, IgA nephropathy, SARS-CoV-2, spinal epidural abscess

## Abstract

A 56-year-old male admitted to the hospital for generalized weakness and fever. He was treated in hospital for 10 days due to COVID-19. He did not receive any immunosuppressive therapy during admission. One day after his discharge he experienced back pain and received analgesic therapy for 10 days. About one month later he experienced severe back pain and gross hematuria. He was admitted to hospital with acute kidney injury and new-onset lower extremity muscle weakness. His renal biopsy revealed IgA nephropathy and thoracic/cervical/lumbar-spine imaging showed an epidural abscess. This is a unique case report of a patient developing an epidural abscess and acute kidney injury together as a serious complication of COVID-19 infection.

Although COVID-19 preferably affects the respiratory systems, some patients present with neurological symptoms such as loss of smell and taste. Guillain–Barré syndrome and acute inflammation of the brain, spinal cord and meninges were also reported [1]. Based on recent studies, acute kidney injury (AKI) is a well-documented complication of COVID-19 and associated with poor prognosis. In this paper we report a case presented with epidural abscess and IgA nephropathy with AKI. The aim of our report is to provide our experience with people who are experiencing multiple and serious complications after COVID-19 infection.

## Case presentation

**Figure 1.**
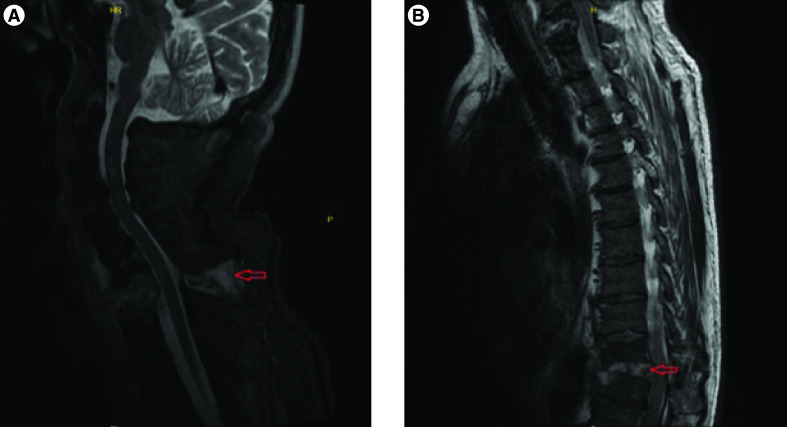
MRI thoracic and cervical spine with contrast. **(A)** Lesions compatible with epidural abscess are observed in C7–T1 bodies (arrow). **(B)** Lesions compatible with epidural abscess are observed in T10–11 bodies (arrow). C: Cervical spine; T: Thoracic spine.

The patient is a 56-year-old man with no prior medical history. He had a history of being treated for COVID-19 one month earlier. His creatinine level was 0.87 mg/dl as normal during that time. During COVID-19 treatment, computed tomographic (CT) angiography was performed to rule out pulmonary embolism because of the low oxygen saturation of the patient. After treatment for COVID-19, he was discharged and at that time he used multiple types of non-steroidal anti-inflammatory drugs (NSAIDs) for his back pain. About one month after his discharge he was admitted to the hospital with gross hematuria and severe back pain. The patient was afebrile at 36.4°C and his blood pressure was high at 166/83 mm Hg. Laboratory findings revealed AKI with serum creatinine 4.83 mg/dl (reference: 0.7–1.3 mg/dl), anemia and lymphopenia. The laboratory values of the patient are summarized in Table 1. A nasopharyngeal sample tested for COVID-19 using reverse transcription PCR (RT-PCR) was negative. Urinalysis revealed with; >363 red blood cells and 46 white blood cells per high-power field and spot urine protein-to-creatinine ratio 2265 mg/g, while ultrasound revealed normal-sized kidneys with grade 2 increased echogenic parenchyma. Serum albumin was low at 2.9 g/dl (reference: 3.2-4.8 g/dl). Serologic examinations for HBV, HCV and HIV were negative. Serum complements (C3 and C4), antinuclear antibodies, anti-extractable nuclear antigen antibodies, antineutrophil cytoplasm antibodies, anti glomerular basement membrane antibodies and anti double stranded antibodies were all unremarkable. Serum protein and immunofixation electrophoresis were negative. Blood and urine cultures grew coagulase-negative *Staphylococcus aureus*. A chest CT scan revealed a 13 mm pericardial effusion and there was no active infiltration detected in the lungs. MRI of cervical–thoracic–lumbar spine with contrast showed circumferential enhancement around the C7–T1 and T10–11 vertebral bodies, and moderate anterior-posterior epidural enhancement with cord-compression consistent with epidural abscess and spondylodiscitis (Figure 1A & B).

**Table 1.** Laboratory values of the patient.

	Day 0	Day 8	Day 12	Day 45	Day 46	Day 53	Day 65	Day 70
Creatinine (mg/dl)	0,87	1.02	0.94	4.83	4.78	6.86	5.57	3.64
GFR (ml/dk/1.73 m^2^)	94	82	83	12	13	8	11	18
Leukocytes (× 10–9/l)	4.6	3.6	7.9	8.72	10.01	8.17	4.3	9.9
Lymphocytes (× 10–9/l)	0.81	1.22	0.67	1.07	0.83	1.16	0.68	1.87
Neutrophils (× 10–9/l)	2.5	2.2	6.84	7.02	8.39	6.12	3.41	7.12
Platelets (× 10–9/l)	210	201	351	401	329	333	305	382
Hemoglobin (g/dl)	15.13	15.36	14.43	10.3	8.7	7.3	6.9	6.9
d-dimer (mg/l)	1.2	0.72	0.28	4.8	4.2	7.9	–	4
CRP (g/l)	0.056	0.074	0.067	0.177	0.152	0.09	0.059	0.01

Day 0: the patient was hospitalized for COVID-19 infection.

Day 12: first day following the patient’s discharge after treatment for COVID-19 infection.

Day 45: the patient was hospitalized for AKI.

AKI: Acute kidney injury; GFR: Glomerular filtration rate.

He was treated with teicoplanin and ciprofloxacin due to epidural abscess and positive blood and urine culture. The patient was consulted with neurosurgery and surgical operation or drainage was not considered. To rule out tuberculosis, purified protein derivative of tuberculin and quantiferon tests were performed and the tests were negative as a result. Renal biopsy was performed; nine glomeruli of 32 samples were involved with a small cellular crescent and fibrinoid necrosis. In immunofluorescent microscopy, mesangial and segmental peripheral capillary wall staining for IgA and IgG were 2^+^ and 1^+^, respectively. There was trace staining for IgM. In addition, 2^+^ staining was observed in the mesangial and peripheral capillary walls for C3c, while no staining was observed for C1q. There is a mixed type of inflammation and tubulitis morphology with diffuse distribution in the interstitium, including eosinophils and neutrophils. The biopsy demonstrated focally crescentic and fibrinoid necrotizing IgA nephropathy with focal mesangial hypercellularity and tubulointerstitial nephritis. Due to an active infectious state, only steroid treatment was planned for the patient. He was consulted with the infectious disease department for rituximab or cyclophosphamide. Infectious diseases approval could not be obtained with active infection. He was treated with methylprednisolone at dose of 1 mg/kg/day. The patient underwent intermittent hemodialysis during the follow-up, serum creatinine decreased and stabilized at 3.6 mg/dl at the end of one month. He consulted with the neurosurgery again and also with the physical therapy department. A corset was recommended for the patient and he was included in the physical therapy program. The patient’s clinic and symptoms improved under antibiotic treatment and physical therapy. In control MRI of the spine showed marked regression of the formation of abscess.

## Discussion

In this report we present a case of IgA nephropathy and epidural abscess together in association with COVID-19 infection. While most of the papers of AKI related to COVID-19 are in hospitalized and in critically ill patients, our case is late onset and with spinal abscess. Although acute tubular injury is the most common renal pathology in kidney biopsy series, recently, an increasing number of patients with glomerular involvement have been reported. These included collapsing glomerulopathy, minimal change disease, membranous glomerulopathy, anti-GBM nephritis, pauci-immune crescentic glomerulonephritis and thrombotic microangiopathy [2–4]. In the autopsy series of 42 patients who died from COVID-19 and AKI, only one patient developed collapsing focal segmental glomerulosclerosis and IgA nephropaty was reported in another with chronic liver disease [5]. Two cases of IgA vasculitis in the course of COVID-19 have been also reported in the literature [6,7].

The mechanisms underlying COVID-19-associated kidney disease are not fully understood and are thought to be very diverse, including direct role of the virus on the renal parenchyma, cytokine storm, multiorgan dysfunction, drug-associated nephrotoxicity and secondary infection with other viruses, bacteria and fungi [2]. COVID-19 also may trigger or exacerbate the immune system and contribute to the immune complex-mediated glomerulonephritis.

In pathogenesis of IgA nephropathy, both glycosylation aberrance of IgA and antibodies directed against galactose-deficient IgA activate formation of immune complexes. Circulating IgA antibodies often have reactivity against antigens from extrinsic microorganisms. Viral infections contribute to the production of these antibodies, and are named as ‘second hits’ in pathogenesis.

Many neurological complications due to COVID-19 such as headache, neuralgia, encephalopathy [8], acute cerebrovascular diseases and cognitive symptoms were also seen [9]. Soh *et al.* first described a case with epidural abscess as a complication of COVID-19, but in their report, it was originating from abscess located in the base of the lung [10].

In humans, ACE2 receptors are widely expressed in regions that have an important role in physiological function, such as the airways, lung parenchyma, vascular endothelium, small intestine and central nervous system [11]. The spike protein of SARS-CoV-2 uses ACE2 as a receptor to gain entrance into the cells [12]. ACE2 is broadly expressed in the apical brush borders of the proximal tubules and also in the podocytes in kidneys [13]. In our report the patient had both IgA nephropathy and epidural abscess together. Although the pathway is not clear, it can be thought that COVID-19 infection causes these complications by using ACE2 receptors [11,14]. In addition, coagulase-negative *S. aureus* growth was observed in the blood and urine cultures of the patient in our case. Therefore, it can be considered that the bacterial infection developing after COVID-19 spreads by hematogenous way and can be the cause of these complications. Another pathway especially for epidural abscess development, may be the infectious process caused by the immunosuppressive treatments taken during COVID-19 treatment. Gianluca *et al.* in their case report, evaluated that spinal cord dysfunction seen in patients after COVID-19 may be related to tocilizumab treatment [15]. But in our report the patient did not take any immunosuppressive treatment when he was hospitalized during COVID-19. Another mechanism that can cause these complications may be endothelial dysfunction caused by COVID-19. Talamonti *et al.* evaluated that epidural abscess may be developed due to endotheliitis caused by COVID-19 [16]. In addition, ACE2 is highly expressed in arterial and venous endothelial systems and these tissues can be directly targeted by COVID-19, which may cause endothelial dysfunction, leading to IgA nephropathy and epidural abscess, as in our case. We think that both of these two complications that were not directly related to each other, were only seen as coincidental. Some of these explanations are endotheliitis, prolonged lymphopenia, ACE2 receptors expressed in central nervous system and kidneys, susceptibility to thrombosis and immune system dysregulation caused by COVID-19 infection [15–18].

It is also known that lymphopenia and immune system dysregulation was developed in COVID-19 [19,20]. In our report, the patient was lymphopenic during COVID-19 treatment and his lymphocyte level returned to normal level before discharge. The lymphocyte course of the patient could not be observed properly for one month after discharge, and it was observed that lymphopenia started again one month later when the patient was admitted to the hospital. It should be considered that the present complications in the patient who was lymphopenic during the development of existing complications may be caused by lymphopenia or immune system dysregulation. As we know, the development of epidural abscess, especially due to *S. aureus* also occurs mostly in immunosuppressed patients, and in our case, COVID-19 seems to be the only reason that can cause immune system dysregulation [21]. A similar situation is observed in HIV infection. This brings to our minds that with a mechanism similar to that in HIV infection, various complications may develop in COVID-19 due to immune system dysregulation [22].

## Conclusion

To our knowledge, there are not many reports of IgA nephropathy and epidural abscess together as a complication. Although, the exact pathway is not clear, in this case report, we wanted to show the possible place of ACE2 receptors and immune system dysregulation in neurological and renal involvement during or after COVID-19 and we also wanted to show healthcare professionals that COVID-19 can present with many complications after discharge.

We hope that this report helps healthcare professionals to be aware of the heterogenous neurological and renal complications of COVID-19. Clinicians must be alert to potential complications during or after the diagnosis and treatment of patients with COVID-19.

It is important to consider the underlying glomerular disease and to exclude rapidly progressive glomerular nephropathies by performing a kidney biopsy when necessary, and clinicians must be alert for neurological symptoms for a better understanding and management of COVID-19 and its complications.

**Executive summary**

- In addition to the common complications in COVID-19, identifying unusual and late onset complications of the disease was thought to be an important measure and would be more helpful in improving clinical management.
- We present a patient who developed IgA nephropathy and epidural abscess concurrently after treatment for COVID-19. In our opinion, immune dysregulation, endotheliitis and secondary bacterial infections, which may be caused by COVID-19, were effective in the development of these complications in the patient.
- It must be kept in mind that the complications caused by COVID-19 can be various and can be seen in the late period.
